# Matrix stiffness promotes cartilage endplate chondrocyte calcification in disc degeneration via miR-20a targeting ANKH expression

**DOI:** 10.1038/srep25401

**Published:** 2016-05-04

**Authors:** Ming-Han Liu, Chao Sun, Yuan Yao, Xin Fan, Huan Liu, You-Hong Cui, Xiu-Wu Bian, Bo Huang, Yue Zhou

**Affiliations:** 1 Department of Orthopedics, Xinqiao Hospital, Third Military Medical University, Chongqing, People’s Republic of China; 2 Institute of Pathology and Southwest Cancer Center, Southwest Hospital, Third Military Medical University, Chongqing, People’s Republic of China; 3 Key Laboratory of Tumor Immunopathology of Ministry of Education of China, Third Military Medical University, Chongqing, People’s Republic of China

## Abstract

The mechanical environment is crucial for intervertebral disc degeneration (IDD). However, the mechanisms underlying the regulation of cartilage endplate (CEP) calcification by altered matrix stiffness remain unclear. In this study, we found that matrix stiffness of CEP was positively correlated with the degree of IDD, and stiff matrix, which mimicked the severe degeneration of CEP, promoted inorganic phosphate-induced calcification in CEP chondrocytes. Co-expression analysis of the miRNA and mRNA profiles showed that increasing stiffness resulted in up-regulation of miR-20a and down-regulation of decreased ankylosis protein homolog (ANKH) during inorganic phosphate-induced calcification in CEP chondrocytes. Through a dual luciferase reporter assay, we confirmed that miR-20a directly targets 3′-untranslated regions of ANKH. The inhibition of miR-20a attenuated the calcium deposition and calcification-related gene expression, whereas the overexpression of miR-20a enhanced calcification in CEP chondrocytes on stiff matrix. The rescue of ANKH expression restored the decreased pyrophosphate efflux and inhibited calcification. In clinical samples, the levels of ANKH expression were inversely associated with the degeneration degree of CEP. Thus, our findings demonstrate that the miR-20a/ANKH axis mediates the stiff matrix- promoted CEP calcification, suggesting that miR-20a and ANKH are potential targets in restraining the progression of IDD.

Low back pain is a leading cause of work-related disabilities worldwide and results in significant health care-related costs^1^. A major cause of low back pain is the intervertebral disc degeneration (IDD)^2^,^3^. Though many factors are associated with IDD, nutritional supplementation plays a crucial role because intervertebral disc (IVD) is the largest avascular organ in the body^4^. Diffusion through the cartilage endplate (CEP) is the major way of obtaining IVD nutrients from the blood supply. The degeneration of CEP is characterized by increased calcification, which decreases the availability of nutrients and exchange of metabolites^5^, resulting in irreversible and progressive IDD^6^,^7^. Therefore, the mechanisms underlying CEP calcification urgently need to be explored.

A series of changes in extracellular matrix (ECM) remodeling, altered solute transport and mineral deposits associated with disturbed inorganic phosphate (Pi) metabolism have been observed with the degeneration of CEP^8^. Pi availability and uptake by chondrocytes play crucial roles in cartilage calcification^9^. Pi levels also increase during IDD, and they are used as indicators of calcification potential^10^.Calcification is associated with mechanical tension and is also regulated by tissue mechanics^11^. Previous studies have demonstrated that the biological effects of matrix stiffness on the proliferation, biosynthetic activity, the maintenance of phenotype, and differentiation of chondrocytes^12^,^13^,^14^. However, the potential pathophysiologic role of matrix stiffness in modulating CEP calcification during IDD has not been reported.

MicroRNAs (miRNAs) belong to a family of non-coding small RNAs composed of approximately 22 nucleotides that bind to the 3′-untranslated regions (UTRs) of their target genes, thereby blocking translation by suppressing expression of or degrading mRNA. Multiple miRNAs have been identified to participate in the cellular response to matrix stiffness, and regulate chondrogenesis, the behavior of MSCs on microgrooved surface patterns^15^,^16^. However, the effects of matrix stiffness on miRNAs expression and, if any, their functional roles in mechanotransduction in CEP chondrocytes have not been well characterized and are, therefore, particularly interesting to be elucidated.

Previous studies have shown that progressive ankylosis protein homolog (ANKH), a multipass transmembrane protein, exports of intracellular inorganic pyrophosphates (PPi) and contributes to the pathophysiology of chondrocalcinosis^17^. ANKH is known to be involved in the local control of mineralization in tissues such as bone, cartilage and in the calcified zone of the growth plate^18^. The baseline expression of ANKH serves to prevent mineral formation under physiologic conditions. Dysregulation of ANKH contributes the formation of calcium pyrophosphate (CPPD) crystals or basic calcium phosphate (BCP) crystal formation^19^. In the current study, we show for the first time that matrix stiffness of human CEP samples is positively correlated with IDD. With co-expression analysis of miRNA and mRNA profiles, we identified a mechanically regulated miRNA, miR-20a, that directly targets (ANKH), an endogenous inhibitor of calcification, to promote stiff ECM-dependent calcification with the elastic modulus corresponding to severe degenerated CEP. In CEP samples, the level of ANKH expression was negatively correlated with the degree of CEP degeneration. This study lends insight into the potential roles of miR-20a and ANKH in the regulation of mineralization in CEPs, providing a better understanding of the vicious cycle of tissue mechanics in the process of CEP degeneration.

## Results

### Degeneration of CEPs is accompanied by collagen disarrangement and increased elastic modulus

**Figure 1:**
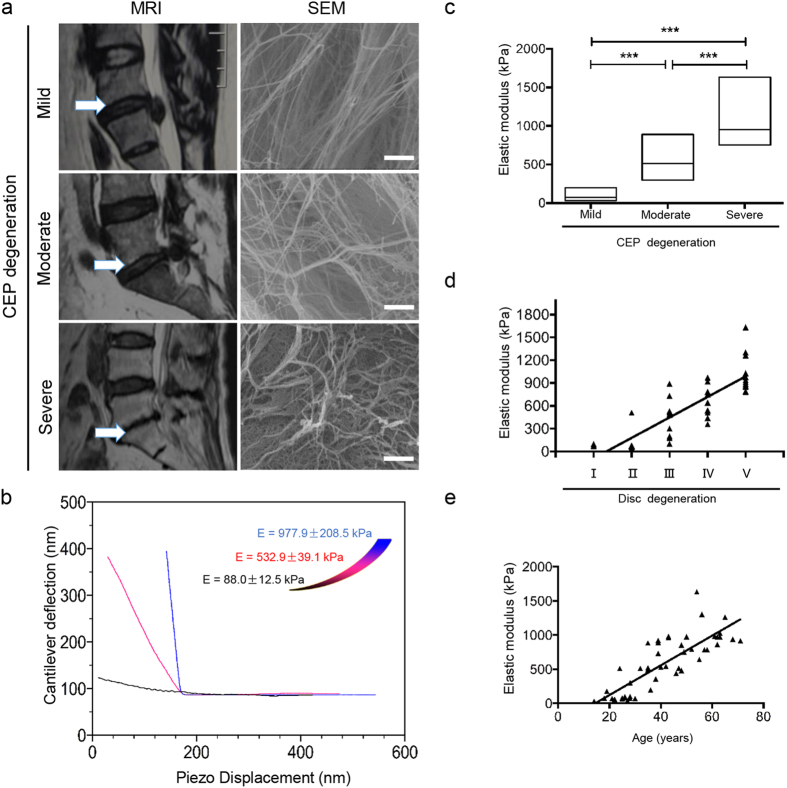
Degeneration of CEPs is accompanied by collagen disarrangement and increased elastic modulus. (**a**) Representative magnetic resonance images (MRIs) of CEPs with mild, moderate or severe degeneration (grades 2, 4, or 6, respectively) obtained from the patients after disc fusion operations. Representative scanning electron microscopy (SEM) image of mild degeneration CEP shows normal collagen meshwork organization. Representative SEM image of moderate degeneration CEP exhibits increased collagen fibril tangles and disarrangement. Representative SEM image of severe degeneration reveals extensive splitting of the collagen meshwork. Scale bars, 0.5 μm. (**b**) Atomic force microscopy indentation tests of CEP samples from the patients. Three average unloading curves of CEPs with different degrees of degeneration (black, mild degeneration, E = 88.0 ± 12.5 kPa; blue, moderate degeneration, E = 532.9 ± 39.1 kPa; red, severe degeneration, E = 977.9 ± 208.5 kPa) show a significant difference in slopes calculated from the curves. (**c**) Elastic modulus of CEPs of different degeneration grades. On each box, the edges and the line of the box are the minimum and maximum data, and the means, respectively. The significant difference in matrix stiffness among the different degeneration degrees of CEPs is shown. (**d**) Correlation between CEP stiffness and the degree of IDD. (**e**) Correlation between CEP stiffness and ages of human patients. Scale bar = 0.5 μm. *indicates P < 0.05, and ***indicates P < 0.001 based on one-way ANOVA.

In order to definitely compare and analyze the matrix stiffness of CEPs with different degrees of degeneration, we collected CEP samples from forty-eight patients after spinal fusion surgery with the degeneration grades of 2, 4, or 6 as classified by the cartilage endplate degeneration classification system^20^ (Grade 2: 14 patients; Grade 4: 14 patients; Grade 6: 20 patients). Patients characteristics are summarized in Supplementary Table 2. Representative magnetic resonance images (MRIs) (Fig. 1a) of the spine showed CEP defects and damage increased with the progression of CEP degeneration, and we designated these degeneration statuses as mild, moderate and severe degeneration, respectively. Scanning electron microscopy (SEM) images (Fig. 1a) of different degenerated CEPs revealed the changes of collagen fibrils and collagen meshwork (in which proteoglycans have been extracted) during the progression of CEP degeneration. SEM images of mild degenerated CEP showed a relatively normal collagen meshwork organization. SEM images of moderate degenerated CEP exhibited increased collagen fibril tangles and disarrangement. SEM images of severe degenerated CEP revealed meshwork disintegration and extensive splitting of the collagen meshwork. It suggested that destabilization of the collagen network increases with the progression of CEP degeneration. A recent study demonstrated that indentation-type atomic force microscopy (AFM) was sensitive to changes in matrix stiffness resulting from early damages to the articular cartilage prior to morphological changes^21^. Similar to articular cartilage, CEP is a layer of hyaline cartilage (approximately 0.6 mm thick) that mainly consists of proteoglycan, collagen and chondrocytes. Therefore, we used AFM method to measure ECM stiffness of CEPs with different degeneration grades. Figure 1b shows the three average loading-displacement curves for CEPs corresponding to different degeneration degrees (mild, moderate and severe degeneration). A significant difference in slopes calculated by the curves shows that the mechanical stiffness (dynamic elastic modulus, E) increased from 88.0 ± 12.5 kPa for mild degeneration to 532.9 ± 39.1 kPa for moderate degeneration to 977.9 ± 208.5 kPa for severe degeneration (Fig. 1c). In addition, we found that matrix stiffness increased with the progression of IDD classified by Pfirrmann grading system^22^ (Fig. 1d) and the elastic modulus of CEP was positively correlated with the degree of IDD (R^2^ = 0.693, P <  < 0.01). Furthermore, the elastic modulus of CEP samples was positively correlated with the age of patients (R^2^ = 0.835, P < 0.01) (Fig. 1e). Combined, these findings suggest that increased ECM stiffness associated with accelerated collagen tangling and identify a relationship between mechanical stiffness, degeneration degree, and the age of patients.

### Matrix stiffness modulates the morphology, cytoskeletal organization and proliferation of CEP chondrocytes

**Figure 2:**
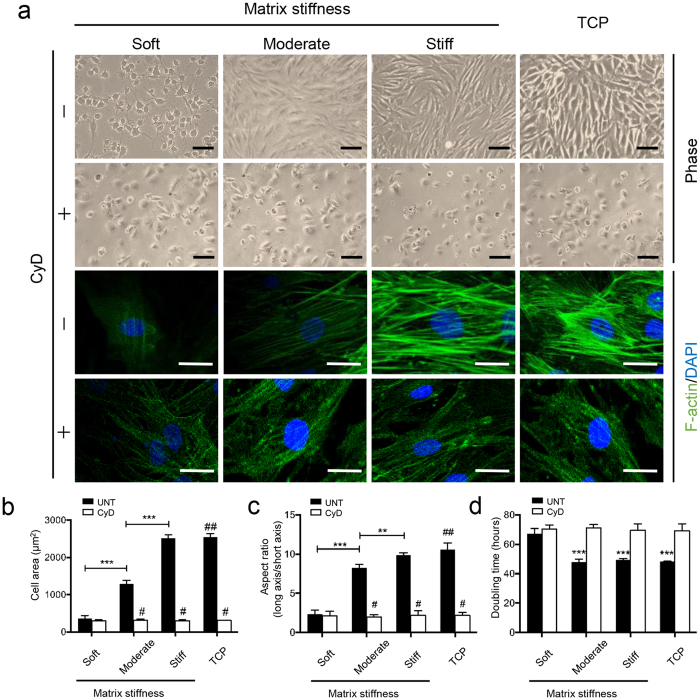
Matrix stiffness modulates the morphology, cytoskeletal organization and proliferation of CEP chondrocytes. CEP chondrocytes were attached and viable on polyacrylamide gels with soft, moderate, or stiff matrix (E’ = 90.0 kPa, 540.0 kPa, and 950.0 kPa, respectively) and compared to those on standard tissue culture plastic (TCP) with or without the addition of cytochalasin-D (CyD) (0.25 μg/mL × 0.5 mL). (**a**) CEP chondrocytes displayed increased cell spreading along with increased matrix stiffness without CyD treatment. In contrast, pharmacologic inhibition of CyD abrogated stiffness-dependent differences in cell morphology. CyD induced rounding and stellated shapes of cells on all substrates. Scale bars, 100 μm (upper two rows), 50 μm (lower two rows). CEP cells areas (**b**) and cell aspect ratio (**c**) were significantly increased in groups with higher stiffness without added CyD. CyD also counteracted stiffness-dependent differences in cell areas and cell aspect ratio. CEP chondrocytes on stiff gels or TCP had the highest aspect ratio and the largest cell area, and those on soft gels had the lowest aspect ratio and the smallest cell area without CyD treatment. UNT, the untreated group. CyD, the group with CyD treatment. ^#^P < 0.001 with respect to the same matrix without added CyD. ^##^P < 0.001 with respect to soft and moderate substrates without added CyD. (**d**) Doubling time of groups with or without added CyD. ***indicates P < 0.001 compared to all other groups. The data are expressed as the means ± SD based on one-way ANOVA.

To further investigate the influence of ECM stiffness on the morphology, cytoskeletal organization and proliferation of CEP chondrocytes, we employed polyacrylamide (PA) gels with varying stiffnesses to simulate mild, moderate and severe degeneration of CEP (corresponding E’ = 90.1 kPa, 540.2 kPa, and 950.7 kPa, respectively) (Supplemental Fig. 1). CEP chondrocytes adopted drastically different morphologies and F-actin organizations in gels with varying stiffnesses and tissue culture plastic (TCP) (Fig. 2a). CEP chondrocytes cultured on soft gels, stiffness similar to that of mild degenerative CEP, maintained a compact, rounded shape. CEP chondrocytes cultured on moderate and stiff gels, which mimicked the moderate and severe degenerative CEP, and the cells cultured on TCP showed increased cell spreading along with increased matrix stiffness. Importantly, cells cultured on a stiff matrix behaved similarly to cells cultured on TCP, suggesting that PA gels do not significantly interfere with adhesion-based cytoskeletal assembly. Cells cultured on progressively stiffer matrices showed increased spreading area (Fig. 2b) and cell aspect ratios (Fig. 2c), with no significant difference between cells cultured on stiff matrix and on TCP. In contrast, pharmacologic inhibition of actin polymerization by addition of cytochalasin-D (CyD) abrogated stiffness-dependent differences in cell morphology and induced rounding and stellated shapes on all substrates (Fig. 2a), which is consist with previous studies^23^,^24^. Proliferation analysis between groups by cell counting showed no significant difference in doubling time among moderate, stiff and TCP groups without the addition of CyD (P > 0.05), but these above were significantly different from the soft group (Fig. 2d). The groups with CyD had significantly longer doubling times than those without CyD. Combined, these data suggest that the biological behaviors of CEP chondrocytes are carefully tuned to the stiffness of the ECM mimicked the degenerative CEP, especially significantly between soft and stiff ECM.

### Matrix stiffness accelerates Pi-induced calcification and regulates the expression of calcification-related genes in CEP chondrocytes

**Figure 3:**
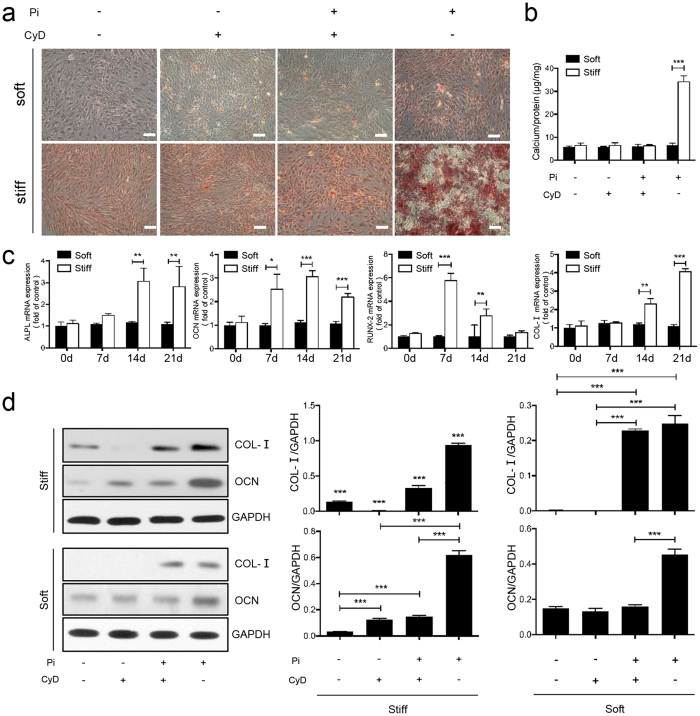
Matrix stiffness accelerates Pi-induced calcification and regulates the expression of calcification-related genes in CEP chondrocytes. (**a**) Observation of calcium deposition in CEP chondrocytes stained by Alizarin red after treatment with or without inorganic phosphate (Pi; 3.0 mmol/L) for 14 days. Scale bars, 100 μm. (**b**) The calcium content assays. (**c**) Reverse transcription (RT)-PCR analysis for the expression of ALP, OCN, RUNX2 and COL-I. The groups on stiff matrix added with Pi showed significantly increased expression of these genes at different time points, compared with the soft group. (**d**) Western blotting shows high COL-I and OCN protein expression on stiff matrix with added Pi. The data are expressed as the means ± SD. *indicates P < 0.05, **indicates P < 0.01, and ***indicates P < 0.001 based on one-way ANOVA.

Calcium deposition is a pathological process related to CEP degeneration that may lead to impairment of nutrient supply and disc metabolism in IVD accompanied by increased levels of inorganic Pi^10^. To explore whether matrix stiffness affected CEP calcification, we examined calcium deposition in CEP chondrocytes cultured on soft and stiff matrices with or without the addition of CyD after treatment with or without Pi (inorganic phosphate; 3.0 mmol/L) for 14 days. As shown in Fig. 3a, stiff ECM alone did not induce calcification in CEP chondrocytes, but it significantly potentiated Pi-induced calcium deposition compared with other groups. As expected, adding CyD inhibited calcium deposition on stiff ECM (Fig. 3a,b). Next, we used quantitative reverse transcription-PCR (qPCR) to analyze the mRNA levels of calcification-related genes at 0, 7, 14, and 21 days (Fig. 3c). The mRNA expression of ALP, OCN, RUNX2, and COL-I was induced by stiff ECM added to Pi without the addition of CyD, and CyD treatment counteracted the expression of calcification-related genes induced by stiff matrix (Supplemental Fig. 2). Consistent with the qPCR results, the protein expression of COL-I and OCN were significantly increased in the stiff ECM group added to Pi (Fig. 3d). These results are consistent with the hypothesis that matrix stiffness modulates calcification and that stiff ECM promotes CEP chondrocyte calcium deposition with addition of Pi.

### Increasing stiffness leads to up-regulation of miR-20a and down-regulation of ANKH in CEP chondrocytes

**Figure 4:**
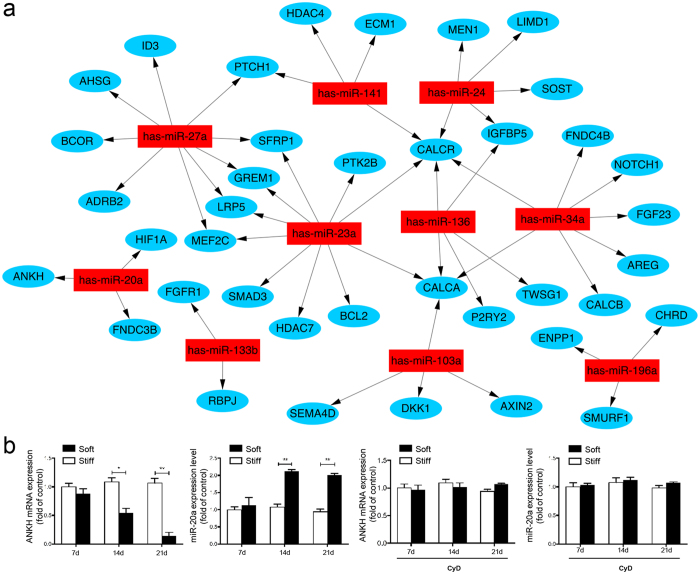
Increasing stiffness leads to up-regulation of miR-20a and down-regulation of ANKH in CEP chondrocytes. (**a**) Bioinformatics analysis based on miRNA and mRNA chips shows a potentially targeting relationship between the most changed miRNA (red) and mRNA (blue) based on miRanda (http://www.microrna.org/) and TargetScan release 6.2 (http://targetscan.org/). (**b**) Validation and quantification of miRNA and mRNA changes using quantitative reverse transcription (RT)-PCR analysis shows significantly reduced ANKH and increased miR-20a in CEP chondrocytes on stiff matrix as compared with the chondrocytes on soft matrix at all days, but there was no significant difference in cells treated with CyD. The level of mRNA and miRNA expression was normalized to GAPDH and U6, respectively and graphed relative to the group cultured on soft matrix. The data are expressed as the means ± SD. *indicates P < 0.05, **indicates P < 0.01, and ***indicates P < 0.001 based on Student’s t-test.

To identify the molecular mechanisms by which CEP chondrocytes respond to differences in the process of calcification when mechanical microenvironment changed, we performed a microRNA and mRNA microarrays expressed in CEP chondrocytes and analyzed the co-expression patterns (Fig. 4a). Four groups of cells were established for the microRNA and mRNA assays: CEP chondrocytes cultured on soft matrix (group 1), moderate matrix (group 2), stiff matrix (group 3) and TCP (control group) for 14 days. The hierarchical clustering results of the miRNA microarray and the mRNA were shown in Supplemental Fig. 3. Interrogation of differentially expressed miRNAs and mRNA showed changes in the expression of 1056 miRNAs and 2780 mRNAs. The top 10 up-regulated miRNAs and the most down-regulated (fold change < 0.5) mRNAs (Fig. 4a) and the top 10 down-regulated miRNA and the most up-regulated (fold change >2) mRNA were separated for construction of a co-expression network. Our prediction that miRNA targets the 3′UTR of mRNA based on an in silico analysis using miRanda (http://www.microrna.org/) and TargetScan release 6.2 (http://targetscan.org/). These findings indicated that miRNAs of CEP chondrocytes were responsive to matrix mechanics and helped us to focus on the process of CEP chondrocyte calcification. Through qPCR validation, we found that the mRNA expression of ANKH decreased and miR-20a expression increased after 14 days on stiff ECM, and CyD abrogated this effect (Fig. 4b). All of these findings indicate that ANKH may be a target of miR-20a.

### miR-20a down-regulates ANKH expression by directly targets the 3′UTR of ANKH mRNA

**Figure 5:**
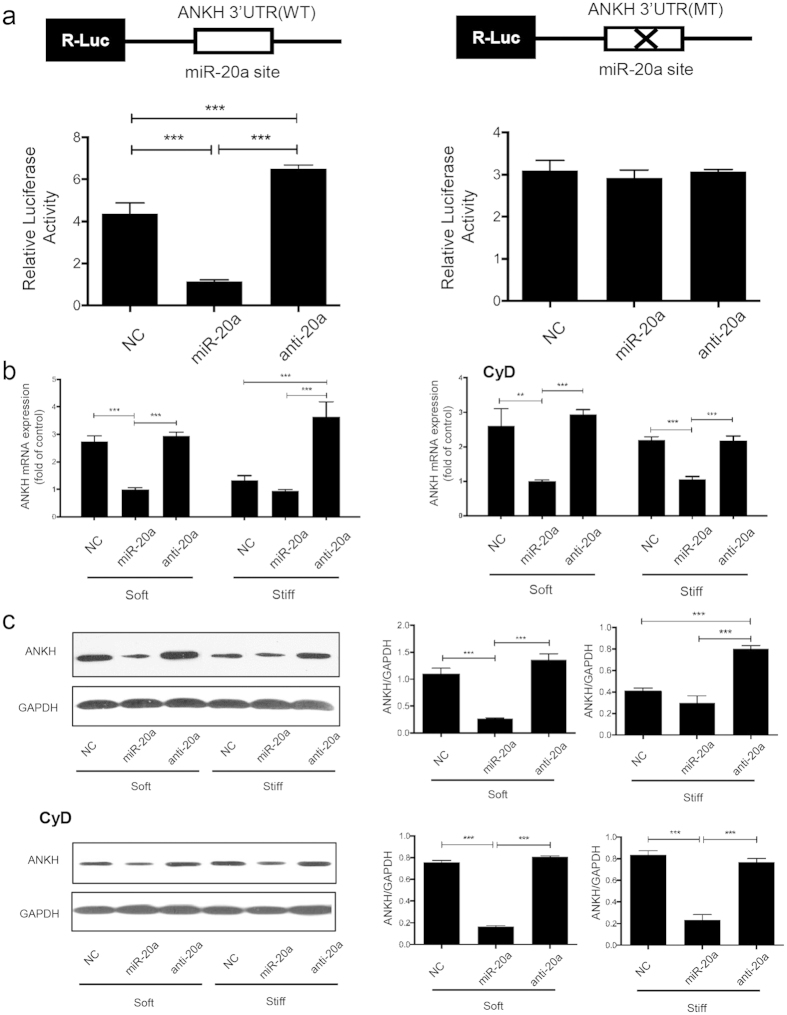
miR-20a down-regulates ANKH expression by directly targets the 3′UTR of ANKH mRNA. (**a**) Dual luciferase assay of the wild-type (WT) group (left panel). Overexpression of miR-20a significantly reduced the relative luciferase activity. Dual luciferase assay of the mutant (MT) group (right panel). No significant differences were observed among the three groups. (**b**) ANKH mRNA expression for CEP chondrocytes cultured on soft or stiff matrix with or without the addition of 0.25 μg/mL CyD for 14 days. The results are normalized to GFPDH and graphed relative to soft matrix. (**c**) Representative western blot images of CEP chondrocytes on soft or stiff matrix with or without the addition of 0.25 μg/mL CyD for 14 days. CyD represents the group with CyD treatment. The data are expressed the means ± SD. *P < 0.05, **P < 0.01, and ***P < 0.001 based on one-way ANOVA.

To validate this prediction, luciferase reporter plasmids (pmirGLO, Promega) containing the full 3′UTR of ANKH (WT) and the mutated miR-20a-binding-site in the 3′UTR of ANKH (MT) were constructed. HEK293 cells were transfected with pre-miR-20a, anti-miR-20a, and a negative control using Lipofectamine 2000. The first group was co-transfected with the WT ANKH plasmid (Fig. 5a, left panel), and the second group was co-transfected with the MT ANKH plasmid (Fig. 5a, right panel). The results revealed that the normalized luciferase activity was significantly decreased in cells transfected with pre-miR-20a compared to the NC group and the anti-miR-20a group with co-transfected with the WT plasmid (P < 0.001; Fig. 5a, left panel). No significant differences were observed between the groups co-transfected with the MT plasmid (Fig. 5a, right panel).

To further verify whether miR-20a directly targets ANKH, we performed qPCR and western blot analyses of ANKH in CEP chondrocytes cultured on soft and stiff matrix for 14 days. Using qPCR, we found that on stiff ECM, the expression of ANKH mRNA was slightly decreased in miR-20a group (p > 0.05), and significantly increased in anti-20a group (p < 0.05), compared with NC group. Similarly, a reduction in the expression of ANKH mRNA after overexpressing miR-20a and an increase of the expression of ANKH mRNA after knocking down miR-20a were observed in the cells cultured on soft matrix (Fig. 5b, left panel). These results indicated that miR-20a repressed translation by degrading ANKH mRNA levels instead of suppressing expression. Interestingly, stiff ECM did not affect the mRNA expression of ANKH when CyD was added (Fig. 5b, right panel), but ANKH mRNA expression was reduced when miR-20a was increased and increased when miR-20a was inhibited. Using western blot analyses, we found that ANKH protein expression was strongly inhibited in the cells transfected with pre-miR-20a on both soft and stiff ECM (Fig. 5c). Consistently, when CyD added, ANKH protein levels were inhibited when miR-20a was overexpressed and were increased when miR-20a was inhibited. CyD abrogated the inhibition of the ANKH protein levels by stiff ECM. Notably, antagomir-mediated silencing of miR-20a restored ANKH levels in CEP chondrocytes grown on soft ECM and caused an overexpression of ANKH on stiff ECM. These findings suggested that ANKH is a direct target of miR-20a in stiff ECM-induced calcification with Pi added.

### Silencing of miR-20a inhibits calcification in CEP chondrocytes on stiff matrix

**Figure 6:**
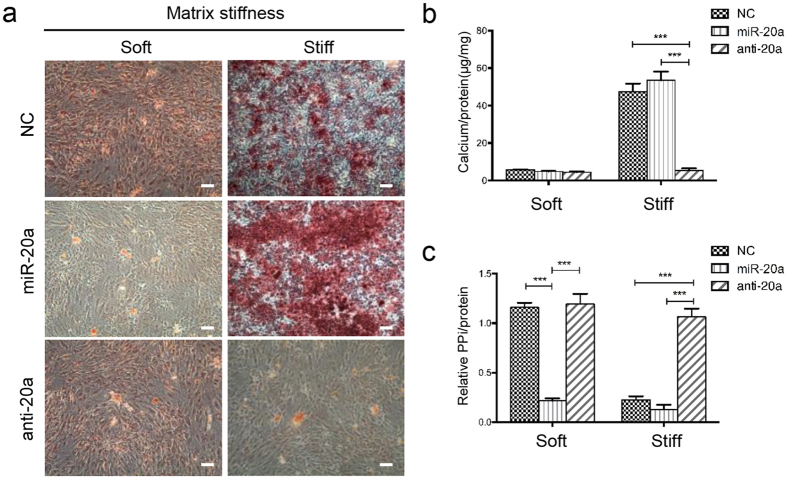
Silencing of miR-20a inhibits calcification in CEP chondrocytes on stiff matrix. (**a**) Representative images of Alizarin red staining of CEP chondrocytes transfected with a negative control (NC), miR-20a, anti-miR-20a using Lipofectamine 2000 and the chondrocytes treated with inorganic phosphate (Pi; 3.0 mmol/L) for 7 days. Scale bars, 100 μm. (**b**) The calcium content assays of the CEP chondrocytes cultured on soft or stiff matrix transfected with NC, miR-20a, or anti-miR-20a. (**c**) PPi levels in the medium of the chondrocytes cultured on soft or stiff matrix transfected with NC, miR-20a, or anti-miR-20a were measured. The data are expressed as the means ± SD. ***indicates P < 0.001 based on one-way ANOVA.

To examine the effect of miR-20a on stiff matrix-induced calcification, CEP chondrocytes transfected with the negative control, pre-miR-20a, or anti-miR-20a, using Lipofectamine 2000 and subsequently cultured on soft and stiff ECM with Pi added for 14 days were subjected to Alizarin red staining (Fig. 6a). All groups on soft ECM did not induce mineralization in CEP chondrocytes (Fig. 6a,b). The NC group and the group overexpressing miR-20a induced mineralization in CEP chondrocytes on stiff ECM, and the calcification of the group overexpressing miR-20a is slightly more than the NC group (p > 0.05). In contrast, CEP chondrocytes transfected with anti-miR-20a showed significantly decreased mineralization with compared to the group transfected with miR-20a and the negative control group (P < 0.001). Calcium deposition results from imbalance of the extracellular levels of Pi, a promoter of calcification, and PPi, an inhibitor of calcification. Therefore, we measured extracellular PPi levels in CEP chondrocytes cultured on soft and stiff ECM. As shown in Fig. 6c, the NC group and the anti-20a group showed high levels of PPi, and the miR-20a group showed significantly decreased PPi secretion in CEP chondrocytes cultured on soft ECM. In contrast, on stiff ECM, the NC group and miR-20a group showed significantly decreased PPi levels, and the anti-20a group restored the high level of PPi (Fig. 6c). Based on these findings, we concluded that silencing of miR-20a inhibits stiff ECM-induced calcification, and the miR-20a target, ANKH, may be involved in this process.

### ANKH regulates calcification in CEP chondrocytes on stiff matrix and is decreased in human degenerative CEP tissues

**Figure 7:**
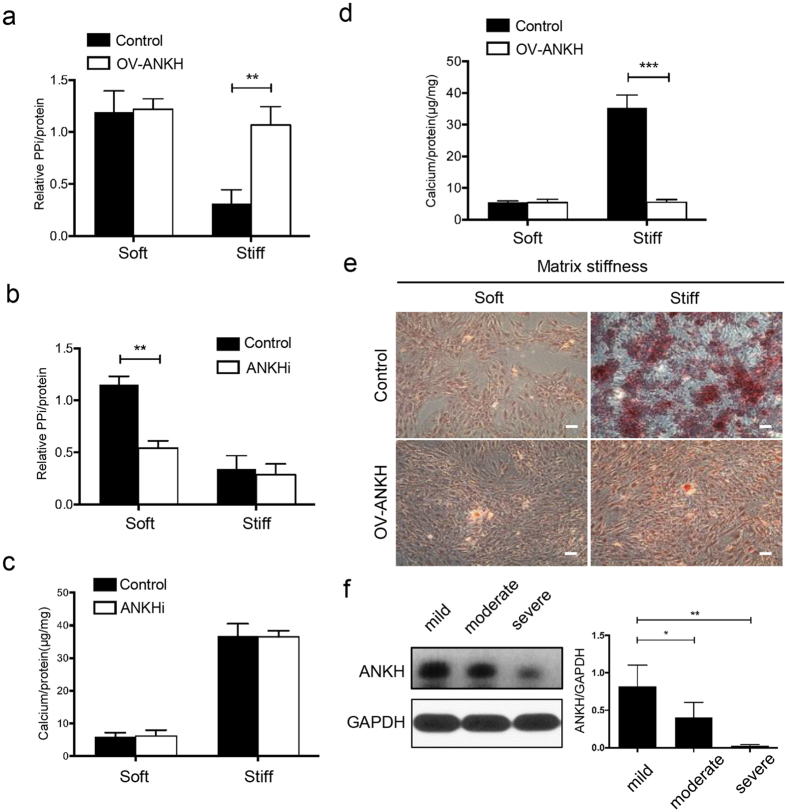
ANKH regulates calcification in CEP chondrocytes on stiff matrix and is decreased in human degenerative CEP tissues. (**a**) PPi levels in the medium of the CEP chondrocytes containing empty vector (Control) or ANKH expression vector (OV ANKH) were measured. (**b**) PPi levels in the medium of the CEP chondrocytes containing empty vector (Control) or ANKH inhibitor (ANKHi, probenecid, 2 mmol/L) cultured on soft or stiff matrix for 14 days were measured. (**c**,**d**) Calcium content assay of the groups treated with OV-ANKH or ANKHi. (**e**) Alizarin red staining showed the protective effect of ANKH overexpression on calcification induced by inorganic phosphate (Pi, 3.0 mmol/L) for 14 days on soft or stiff matrix. Scale bars, 100 μm. (**f**) Representative western blot images of ANKH in human CEP samples obtained from patients undergoing disc fusion operations. The expression level of ANKH decreased along with the degenerative degrees of human CEP. The data are expressed as the means ± SD. *indicates P < 0.05, **indicates P < 0.01, and ***indicates P < 0.001 based on one-way ANOVA.

To determine whether stiff ECM induces CEP calcification by inhibiting ANKH expression, we examined whether ectopic expression of ANKH could inhibit stiff ECM-induced CEP calcification in CEP chondrocytes by retroviral transduction (Supplemental Fig. 4). On soft ECM, PPi secretion remained high level and was not significantly different between CEP chondrocytes infected with an empty vector (control group) and the ANKH expression vector (OV-ANKH) (Fig. 7a). In contrast, ANKH overexpression significantly restored PPi secretion compared with the control group on stiff ECM (Fig. 7a), and an ANKH inhibitor (probenecid, 2 mmol/L) significantly decreased PPi secretion on soft ECM (Fig. 7b). Further, we performed Alizarin red staining and calcium content assays of the control group and the OV-ANKH group on soft and stiff matrices (Fig. 7d,e), and we found that soft matrix did not induce calcium deposition on control group or OV-ANKH groups. Stiff matrix induced calcification in the control group. But overexpression of ANKH reversed the induction of calcification by stiff ECM, thus providing evidence that stiff ECM mediates the induction of CEP chondrocyte calcification via targeting ANKH. Representative western blot images of ANKH in human CEP samples obtained from patients undergoing disc fusion operation are shown (Fig. 7f). The expression level of ANKH decreased accompanied with the degeneration degree of CEP in human patients. These findings suggest that stiff matrix may induce CEP chondrocyte calcification by downregulating the expression of ANKH and PPi secretion.

## Discussion

The etiology of IDD is related to various factors, including hyper-physiological loading, abnormal MMPs and inflammatory cytokines. Much of the recent research on IDD has focused on the functional restoration of IVD via gene therapy, growth factors, and tissue engineering. However, the underlying mechanisms of IDD remain largely unknown. Recent reports of altered mechanics regulating tissue behavior have provided new insight into the pathophysiology of IDD. CEPs are known to play a crucial role in biomechanical integrity and IVD nutrition. CEP degeneration directly alters ECM composition and increases mineralization, resulting in decreased nutrient supply to the disc. Remodeling of this ECM with degeneration or age may develop into a vicious cycle resulting in CEP calcification, whereby altered tissue mechanics may induce calcification in the surrounding ECM. To define a correlation between pathophysiologically relevant stiffness and CEP degeneration, it is necessary to measure tissue stiffness of CEP with different degree of degeneration. For endplate changes, traditional scoring system is Modic scale^25^, which mainly appear to reflect a spectrum of vertebral body marrow and subchondral bone changes, but could not clearly depict the status of the cartilage endplate. Rajasekaran *et al.*^20^ described the cartilage endplate degeneration classification which classifies the cartilage endplate degeneration into six types, involving in considering cartilage endplate damages of varying severity. Although Modic scale is the validated scoring system, Rajasekaran’s cartilage endplate degeneration classification is more accurate in considering the changes of morphology and damage of cartilage endplate. Since the changes of morphology and damage of cartilage endplate are the main topic, the Rajasekaran’s method is more appropriate to our study. For mechanical measurement, AFM-based approaches are attractive for assessing mechanical properties of biological tissues because they quantify structural and mechanical characteristics^26^,^27^,^28^. To the best of our knowledge, AFM has not previously been employed to measure the mechanical properties of human CEP tissues. We used indentation-type AFM to investigate elastic responses of human CEP tissues with different degeneration degrees. We demonstrate that the elastic modulus of CEPs is increased with the degree of CEP degeneration, and is positively correlated with the degree of IDD and the age of human patients. It recently showed that age, rather than the presence of osteoarthritis (OA), is the predominant factor driving progressive pathologic calcification in articular cartilage^29^. We subsequently constructed a comparatively simple hydrogel system using polyacrylamide gels to simulate the stiffnesses of CEP tissues with different degeneration degrees and verified the elastic modulus of polyacrylamide gels by AFM with the same parameters. In this study, we used monolayer culture with varying stiffness to mimic the stiffness of human differently degenerated CEP. It is a common model for study on matrix mechanics and allows discrimination between responses of CEP chondrocytes to mechanical and structural changes in the ECM. However, since CEP chondrocytes grow physiologically in a special microenvironment and the monolayer culture can alter the phenotype of CEP chondrocytes, it is necessary to develop a 3-dimensional culture system to avoid this limitation in our further study.

It was recently documented that tissue mechanics strongly influence cell biological behaviors. The changes in tissue mechanics have been associated with many diseases, such as atherosclerosis^30^, fibrosis^31^, and cancer^32^. Matrix stiffness is one of many aspects of tissue mechanics that compromise tissue architecture, microgrooved surface patterns, and interstitial pressure; these physical cues also influence cell biological behaviors. Accumulating evidence suggests that the mechanical cues around cells regulate cell behaviors, including chondrocyte hypertrophy, chondrocyte differentiation, and nucleus pulposus cell-cell interactions and the fate of nucleus pulposus-derived stem cells^33^,^34^,^35^,^36^. Although mechanistic studies of CEP calcification have historically focused on genetic and biochemical factors, we began to explore the role of matrix stiffness in controlling cell behaviors that contribute to calcification. Our study revealed significant stiffness-dependent differences in CEP chondrocyte shape, cytoskeletal organization, and proliferation. Similar to previous studies^24^,^37^, increasing stiffness increased cell spreading and proliferation. Cells respond to matrix stiffness by increasing internal cellular tension through stress fiber formation. In our study, both cell area and aspect ratio increased with matrix stiffening and temper stiffness-dependent differences in cell shape and proliferation by inhibiting actin polymerization. Pharmacologic inhibition of actin polymerization blunted the sensitivity of CEP chondrocytes to matrix stiffness. It suggests the biological behaviors of CEP chondrocytes are sensitive to the stiffness of the extracellular microenvironment. In our study, CEP chondrocyte calcification was precisely tuned to the stiffness of the ECM, such that the maximal calcification occurred on matrix that mimic the stiffness of severe degenerative CEP. Prevention of the stiffness increase of CEP represents a potential target for maintaining spine health and preventing spondylopathy. However, the pathological significance of matrix stiffness and key molecules that regulate this process remain to be elucidated.

Recently, miRNA has attracted attention because it plays a crucial role in human disease and may be a potentially new therapeutic target. It is estimated that miRNAs regulate ~30% of human protein-coding genes, demonstrating the key role of miRNAs in controlling gene expression^38^. Increasing evidence indicates that many cellular processes, including proliferation, calcification and differentiation, are regulated by miRNAs. Therefore, miRNAs have considerable potential to become a focus for prevention and treatment of IDD^39^. Several studies indicated that tissue mechanics including matrix stiffness, could modulate the expression of a panel of miRNAs which are involved in the cellular response to mechanical stiffness in different cell lines^40^,^41^. Studies have shown miRNAs to be an unavoidable regulatory factor in chondrocyte hypertrophy and cartilage calcification^42^,^43^,^44^. However, no miRNA has yet been reported to be involved in the process of matrix stiffness regulating chondrocyte calcification. In this study, we screened for mechano-sensitive miRNAs in stiffness-induced CEP chondrocyte calcification and identified that miR-20a was increased with the increase of matrix stiffness through the co-expression network of microRNA and mRNA microarrays. The inhibition of miR-20a reduced calcium deposition and increased the extracellular PPi levels. We demonstrated that miR-20a directly target ANKH, and thereby inhibits its expression and induces stiff ECM-mediated calcification.

After confirming the direct targeting relationship between miR-20a and ANKH, we focused on the function of ANKH to further elucidate the mechanism of stiff ECM-induced calcification. Extracellular levels of Pi and PPi are major regulators of the calcification process. Four membrane proteins, including ANKH, PiT-1, PC-1 and tissue non-specific alkaline phosphatase (TNAP), control the balance between Pi and PPi. ANKH, a multiple-pass transmembrane protein, exports inorganic PPi from the cytoplasm to inhibit hydroxyapatite formation and prevent mineralization in skeletal tissue^18^. ANKH inhibits calcification by controlling PPi levels. Deletion of *ANKH* in mice results in abnormal calcification in soft tissues and articular cartilage^45^. *ANKH* mutations result in two distinct mineralization diseases: familial calcium pyrophosphate dihydrate deposition disease^46^ and craniometaphyseal dysplasia^47^,^48^. The reports effects of *ANKH* mutations on PPi levels are conflicting, possibly because gain-of-function mutations in *ANKH* are subject to a transcriptional down-regulation, in which Pi and PPi regulate the expression profiles of the genes that control their production^49^. Numerous studies have suggested that extracellular PPi generation by both adenosine triphosphate- and ANKH-dependent mechanisms is key for cartilage and muscle cells calcification^50^,^51^. In our study, stiff ECM markedly repressed ANKH expression and PPi secretion and promoted mineralization. In contrast, we found that soft ECM had no effect on the expression of ANKH or PPi secretion. In addition, the restoration of ANKH expression greatly rescued stiff ECM-induced calcification and PPi secretion. We verified the ANKH expression was decreased with the degree of CEP degeneration in human CEP, which is inconsistent with the increased level of ANKH expression in cartilage from patients with osteoarthritis (OA)^52^. The baseline expression of ANKH serves to prevent mineral formation under physiologic conditions^19^. It might be attributed to the difference in the pathophysiological process and microenvironment between CEP degeneration and OA. Collectively, these data strongly indicate an important role for ANKH involved in the process of CEP calcification. Because our research is limited to *in vitro* studies, further research is needed to validate the function of ANKH *in vivo*.

In conclusion, we found that matrix stiffness was increased with IDD, suggesting a potential role for matrix stiffness in the pathogenesis of IVD degeneration. We demonstrated that the expression of miR-20a was markedly elevated in association with increased stiffness, and miR-20a inhibited ANKH expression by directly targeting the 3′-UTR of ANKH. This inhibition was abolished by overexpression of ANKH. This suggests that the new regulatory miR-20a/ANKH pathway may be an attractive target for therapeutic modalities to treat IVD degeneration.

## Materials and Methods

### Human CEP samples

Human intervertebral disc CEP samples were obtained from totally forty-eight patients (twenty-eight males, twenty female) undergoing spinal fusion surgery in Xinqiao Hospital (Chongqing, China). Magnetic resonance imaging (MRI) scanning of the spine was performed for all the patients prior to surgery. CEP samples were harvested by two experienced spine surgeons (H.B., Y.Z.) according to protocols approved by the Medical Ethical Committee of the Xinqiao Hospital, which comply with the principles outlined in the Declaration of Helsinki. Informed consent was obtained before collection of all samples involved in the study. Institutional review board approval was granted to our study. The average age was 41.5 years (range: 14–71 years), and the male/female ratio was 1.4. Detailed information of the patients is shown in Supplement Table 1.

### Atomic force microscopy (AFM)

For AFM measurements, the surgical CEP samples were firstly prepared as described previously^53^. CEP samples were embedded in Tissue-Tek (Tissue-Tek, 4583 Compound, Netherlands) and sectional with a cryostatic microtome at −15 °C. From the ~5 mm × 5 mm pieces, ~2 mm in thickness, the outermost (~1 mm thick) layer of the CEP surface was discarded to minimize surface irregularities and tilt. Then, 20-mm-thick frozen sections were allowed to adhere to glass coverslips that had been coated with a 0.01% poly-l-lysine solution (Sigma-Aldrich, USA). Secondly, CEP tissues were glued onto a round Teflon disk with a minimum amount of Histoacryl (B. Braun Surgical GmbH, Germany). The mounted samples were kept in PBS (4 °C) until use. We used AFM (Nanowizard II, JPK Instruments, Germany) to measure the matrix stiffness (dynamic elastic modulus, E) of CEP tissues as described previously^54^. Briefly, we recorded force-displacement curves during the process of loading and unloading. Force-displacement curves on CEP tissues were recorded with a sharp pyramidal tip (200-mm-long silicon nitride cantilevers, nominal cantilever spring constant k = 0.06 N/m, Veeco, USA) corresponding to an applied load of ~2 nN and a maximum indentation of ~100–500 nm. Matrix stiffness was probed at 3 Hz, which is similar to human gait. On every CEP tissue sample, 100 points were probed in 1 × 1 cm^2^ sample regions; at least 10 different regions were measured. The Hertz model is only acceptable for incompressible homogeneous and elastic material (Poisson’s ratio = 0.5). However, the elastic modulus is generally accepted as a measure of matrix stiffness of tissues under small strains^55^. All images and data were processed using AFM software (JPK Instruments).

### Scanning electron microscopy (SEM)

SEM detected the collagen structures of CEP samples, as described previously^21^. Briefly, proteoglycans were extracted from CEP samples in 100 mM Soerensen’s phosphate buffer (pH 7.2) (Sigma-Aldrich) containing 1 mg/mL trypsin (Sigma-Aldrich), 1 mg/mL bovine hyaluronidase (Sigma-Aldrich), protease inhibitors (Sigma-Aldrich) and 0.01% NaN_3_ (Sigma-Aldrich) at 37 °C for 3 days. The samples were fixed with 2.5% glutaraldehyde (Sigma-Aldrich) in PBS and dehydrated in a graded ethanol series. After point drying, the CEP samples were sputter-coated with 3–5 nm platinum and examined with an SEM (S-3400N II-Hitachi, Japan) at 1.5–5 kV accelerating voltage.

### Fabrication of polyacrylamide gels

Cells were plated on variably compliant polyacrylamide gels, according to a previously established protocol^56^. In brief, 12 mm glass coverslips were flamed in a Bunsen burner, soaked in 0.1 mol/L NaOH, and air dried. A small aliquot of 3-aminopropyltrimethoxysilane (Sigma-Aldrich) was spread evenly onto the glass surface. After 5 min, the coverslips were washed and soaked in distilled H_2_O. The coverslips were then immersed for 30 min in a solution of 0.5% glutaraldehyde in PBS buffer. The coverslips were then washed extensively in distilled H_2_O and air dried. Polyacrylamide gel solutions with acrylamide were prepared at final concentrations ranging from 8 to 51 wt/vol% and bis-acrylamide ranging from 0.15 to 3.825 wt/vol%. To polymerize, 5 μL of 10 wt/vol% ammonium persulfate (Sigma-Aldrich) and 1.5 μL TEMED (Sigma-Aldrich) were added with the appropriate amount of PBS to yield a final volume of 250 μL. Gel precursor solution (10 μL) was immediately pipetted onto the silanized glass coverslips, and a 20 mm glass coverslip was placed on top of the polymerization solution. After 2 hours, the samples were soaked in PBS buffer overnight. For cell seeding, rat tail Collagen-I protein (Sigma-Aldrich, USA) was conjugated to the surface of the PA gels using the heterobifunctional linker N-sulfosuccinimidyl-6-(4′-azido-2′-nitrophenylamino) hexanoate (sulfo-SANPAH, Pierce, USA). Thirty microliters of a 1 mg/mL solution in milli-Q H_2_O was pipetted onto the gel surface in a 12-well plate, which was placed under a 365 nm UV LED array (0.8 mW, 20 mA; Cetoni, Germany) and irradiated for 15 min. The gels were washed with 50 mM HEPES in PBS, and the procedure was repeated thrice. Then the gels were coated with 0.25 μg/cm^2^ rat tail Collagen-I protein in PBS for overnight at room temperature. The gels were washed three times with PBS. The stiffness of the PA gels was measured with the same method as described for the CEP tissues above.

### CEP cells isolation and culture

Primary CEP cells were obtained from CEP tissues from six donors (mild degeneration of CEP), as previously described^57^. Briefly, CEP tissues were carefully examined to remove any obvious ligament tissue or granulation tissue. After the CEPs were minced into 1 mm^3^ blocks, CEP cells were isolated by digestion medium containing Dulbecco’s modified Eagle’s medium (DMEM)/F12 (Gibco, Grand Island, NE), 0.5% Trypsin (Gibco), 0.3% type-II collagenase (Gibco) and 1% penicillin-streptomycin (Gibco) at 37 °C for 8 hours. The suspended cells were filtered through a 70-μmcell filter to minimize cell aggregation. The cell suspension was transferred to a 15 mL polypropylene culture tubes and centrifuged for 5 min at 100 × g. The suspension solution was discarded, and the pallet was resuspended in the expansion culture medium containing DMEM/F12, 10% fetal bovine serum (FBS; Gibco) and 1% penicillin-streptomycin. The second passage of CEP chondrocytes were used in our calcification experiments. CEP chondrocytes were collected and plated in culture plates and then cultured in the expansion culture medium or calcification medium with 3.0 mmol/L Pi (sodium phosphate (Na_2_HPO_4_ and NaH_2_PO_4_); Sigma-Aldrich) under a humidified atmosphere containing 5% CO_2_ at 37 °C.

### Microscopy, cytoskeletal organization, morphometric analysis

CEP chondrocytes were cultured on the PA gels of increasing stiffness (73.2 kPa, 512.7 kPa, 978.5 kPa) and TC plastic for 7 days. All live-cell imaging was performed using an inverted Leica microscope equipped with a motorized programmable stage. Cytoskeletal organization within the cells was identified using a fluorescent stain for filamentous actin (F-actin). CEP cells were fixed and stained with Alexa Fluor 488 phalloidin (Invitrogen; diluted 1:500), and the nuclei were stained with 4′,6-diamino-2-phenylindole (DAPI) (Invitrogen; diluted 1:200). Cell spreading area and aspect ratio measurements were obtained by quantifying the area of phalloidin-stained cells using Image J software (NIH). For aspect ratio calculations, the longest pair of perpendicular lines that crossed at the nucleus was drawn. The aspect ratio was then calculated as the length of the longest line in the pair divided by the length of the shortest line.

### miRNA microarray, mRNA microarray, and bioinformatics analysis

CEP chondrocytes were cultured on PA gels of increasing stiffness (E’ = 90.1 kPa, 540.2 kPa, and 950.7 kPa, respectively) and TCP with added Pi (3.0 mmol/L) for 14 days, and each sample was biologically repeated three times. Total RNA was extracted from CEP cells using Trizol (Invitrogen, USA). mRNA and miRNA microarray hybridizations were performed with total RNA using the miRCURYTM LNA Array (v.18.0) (Exiqon, Denmark) in KangChen Bio-tech (Shanghai, China) and Affymetrix Gene Chip Human Gene Array (Affymetrix, USA) in CapitalBio (Beijing, China). After subtracting the background, the fluorescence value was detected. The ratio of the two subgroups (log2-transformed) and the p-values of the t-test were calculated. The mRNAs and miRNAs with a P-value less than 0.05 were selected.

### Dual luciferase reporter assay

The wild-type (WT) 3′-UTR of ANKH containing the miR-20a binding site was cloned into the pmirGLO vector (Promega, USA). The miR-20a complementary site with the sequence GCACTTTA in the ANKH 3′UTR was mutated to TACAGGGC to remove its complementarity to miR-20a. Control HEK293 cells and HEK293 cells transfected with the pre-miR-20a precursor or miR-20a-inhibitor were cultured in 24-well plates (2.5 × 10^4^) and co-transfected with 100 ng of wild-type or mutated ANKH 3′UTR construct. Luciferase activity was measured using a dual luciferase reporter assay system (Promega, USA). Measurements of luminescence were performed with a luminometer (Glomax 20/20, Promega).

### PPi assay

Culture medium PPi levels were measured using the PPiLight Pyrophosphate Detection Kit (Lonza, USA) according to the manufacturer’s instructions. Briefly, 40 μl of medium was added to 20 μl of PPiLight detection reagent, and the initial steady-state bioluminescence was measured after a 10 min incubation at room temperature, as basal level. Then in another tube, 40 μl of medium sample was added to 20 μl of PPiLight converting reagent and incubated for 30 min; then 20 μl of PPiLight detection reagent was addedand incubated for 30 min. Finally, luminescence was read, and the value subtracted from the original bioluminescence value to obtain PPi-dependent bioluminescence.

### Calcification staining and related quantitative assays

For the evaluation of mineralized matrix, cells were fixed with 4% formaldehyde and stained with 1% Alizarin red (Sigma-Aldrich) solution in water for 20 minutes. Then the cells were stained with 0.1% ARS in distilled water (pH 4.2) for 30 minutes. After washing with distilled water 3 times, the cells were incubated with PBS at 37 °C for 15 min. To quantify matrix calcification, ARS-stained plates were incubated with 300 μL 10% CPC (cetylyridinium chloride) at 37 °C for an additional 15 min, and the optical density of the extracted dye was evaluated at 562 nm in a spectrophotometer. The ARS intensity was compared with that of the control treatment and calculated after normalization to the total protein content.

### Statistical analysis

The data are presented as the means ±  SD (standard deviation). The SPSS version 13.0 software (SPSS Inc., IL, USA) was used for statistical analysis. Statistical differences were measured with Student’s t-test for comparison between two groups or analysis of variance (ANOVA) followed by Turkey’s t-test for comparison of multiple groups. The paired-samples t test was used to compare within groups at different time points. P values less than 0.05 were considered to be significant.

## Additional Information

**How to cite this article**: Liu, M.-H. *et al.* Matrix stiffness promotes cartilage endplate chondrocyte calcification in disc degeneration via miR-20a targeting ANKH expression. *Sci. Rep.* **6**, 25401; doi: 10.1038/srep25401 (2016).

## Supplementary Material

Supplementary Information
