# Correction: Matrix stiffness promotes cartilage endplate chondrocyte calcification in disc degeneration via miR-20a targeting ANKH expression

**DOI:** 10.1038/s41598-026-68687-0

**Published:** 2026-09-08

**Authors:** Ming-Han Liu, Chao Sun, Yuan Yao, Xin Fan, Huan Liu, You-Hong Cui, Xiu-Wu Bian, Bo Huang, Yue Zhou

**Affiliations:** 1 Department of Orthopedics, Xinqiao Hospital, Third Military Medical University, Chongqing, People’s Republic of China; 2 Institute of Pathology and Southwest Cancer Center, Southwest Hospital, Third Military Medical University, Chongqing, People’s Republic of China; 3 Key Laboratory of Tumor Immunopathology of Ministry of Education of China, Third Military Medical University, Chongqing, People’s Republic of China

Correction to: *Scientific Reports* https://doi.org/10.1038/srep25401, published online 04 May 2016

This Article contains errors.

Several representative images in Figures 2a and 3a had been inadvertently misplaced during figure assembly. The representative images currently shown do not accurately correspond to the indicated experimental groups because of inadvertent errors during figure assembly. **These corrections do not affect the quantitative results or conclusions of the Article.**

The corrected Figures 2 and 3 are shown below.

**Fig. 2:**
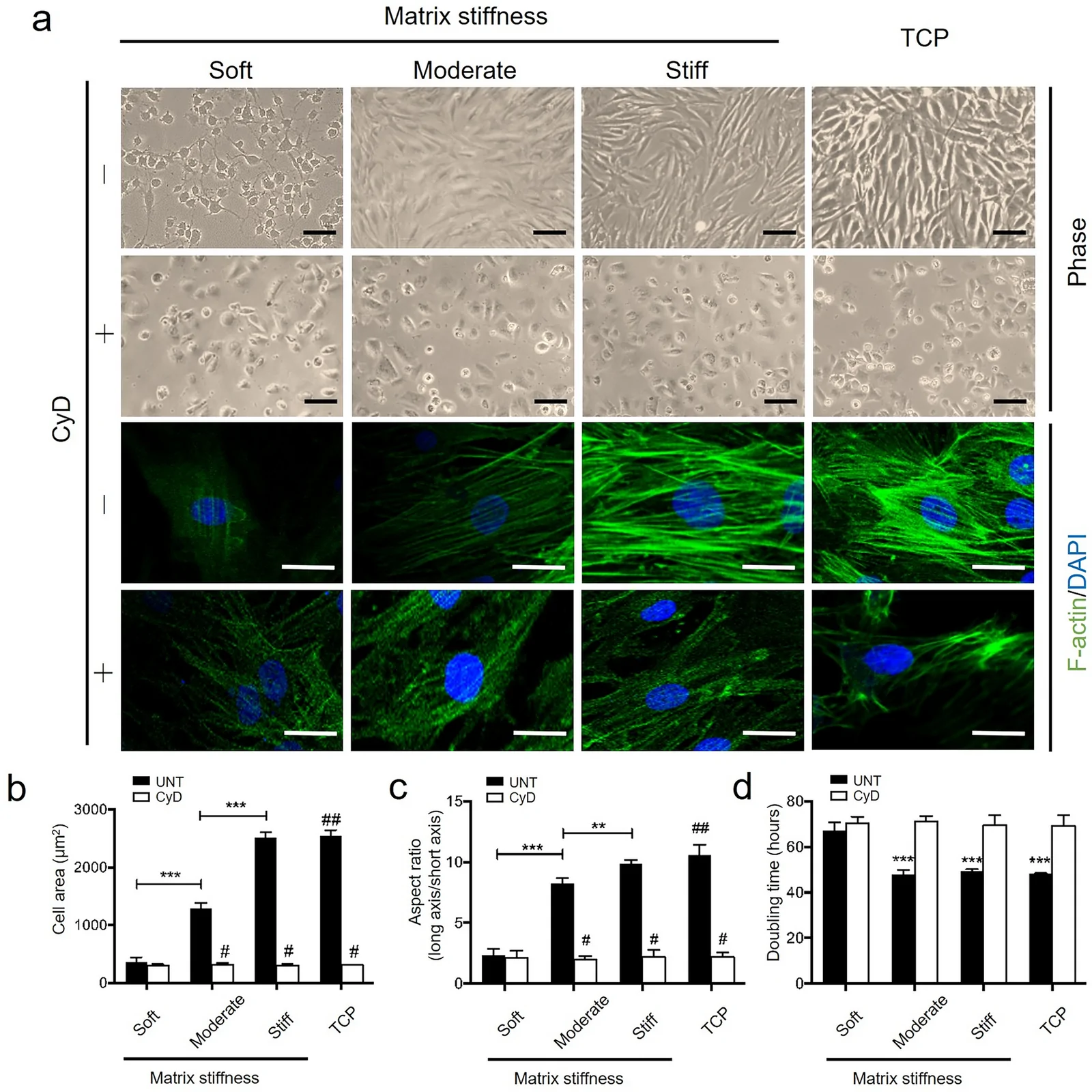
Matrix stiffness modulates the morphology, cytoskeletal organization and proliferation of CEP chondrocytes. CEP chondrocytes were attached and viable on polyacrylamide gels with soft, moderate, or stiff matrix (E’ = 90.0 kPa, 540.0 kPa and 950.0 kPa, respectively) and compared to those on standard tissue culture plastic (TCP) with or without the addition of cytochalasin-D (CyD) (0.25 μg/mL × 0.5 mL). (**a**) CEP chondrocytes displayed increased cell spreading along with increased matrix stiffness without CyD treatment. In contrast, pharmacologic inhibition of CyD abrogated stiffness-dependent differences in cell morphology. CyD induced rounding and stellated shapes of cells on all substrates. Scale bars, 100 μm (upper two rows), 50 μm (lower two rows). CEP cells areas (**b**) and cell aspect ratio (**c**) were significantly increased in groups with higher stiffness without added CyD. CyD also counteracted stiffness-dependent differences in cell areas and cell aspect ratio. CEP chondrocytes on stiff gels or TCP had the highest aspect ratio and the largest cell area and those on soft gels had the lowest aspect ratio and the smallest cell area without CyD treatment. UNT, the untreated group. CyD, the group with CyD treatment. ^#^P < 0.001 with respect to the same matrix without added CyD. ^##^P < 0.001 with respect to soft and moderate substrates without added CyD. (**d**) Doubling time of groups with or without added CyD. ***indicates P < 0.001 compared to all other groups. The data are expressed as the means ± SD based on one-way ANOVA.

**Fig. 3:**
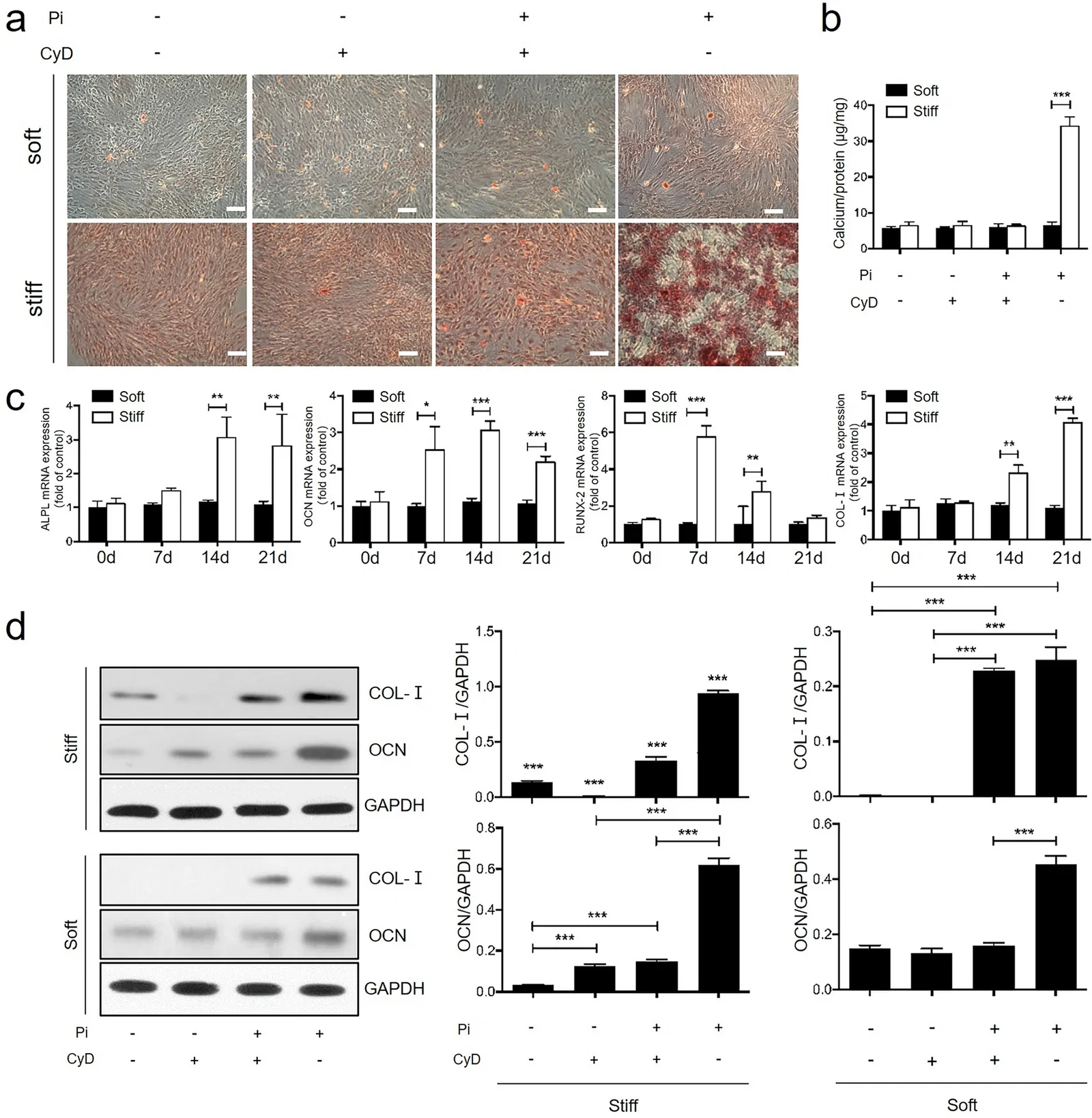
Matrix stiffness accelerates Pi-induced calcification and regulates the expression of calcification-related genes in CEP chondrocytes. (**a**) Observation of calcium deposition in CEP chondrocytes stained by Alizarin red after treatment with or without inorganic phosphate (Pi; 3.0 mmol/L) for 14 days. Scale bars, 100 μm. (**b**) The calcium content assays. (**c**) Reverse transcription (RT)-PCR analysis for the expression of ALP, OCN, RUNX2 and COL-I. The groups on stiff matrix added with Pi showed significantly increased expression of these genes at different time points, compared with the soft group. (**d**) Western blotting shows high COL-I and OCN protein expression on stiff matrix with added Pi. The data are expressed as the means ± SD. *indicates P < 0.05, **indicates P < 0.01 and ***indicates P < 0.001 based on one-way ANOVA.

