# Supplementary material for: Matrix stiffness promotes cartilage endplate chondrocyte calcification in disc degeneration via miR-20a targeting ANKH expression
Source: Sci Rep. 2016 May 4;6:25401. doi: 10.1038/srep25401 (PMC4855171; doi:10.1038/srep25401)
Supplement: Supplementary Information [file srep25401-s1.doc]

**Supplementary information**

**Matrix stiffness promotes cartilage endplate chondrocyte calcification in disc degeneration via miR-20a targeting ANKH expression**

Ming-Han Liu^1^, Chao Sun^1^, Yuan Yao^1^, Xin Fan^1^, Huan Liu^1^, You-Hong Cui^2,3^, Xiu-Wu Bian^2,3^, Bo Huang^1,*^, Yue Zhou^1,*^

*^1^Department of Orthopedics, Xinqiao Hospital, Third Military Medical University, Chongqing 400037, China*

*^2^Institute of Pathology and Southwest Cancer Center, Southwest Hospital, Third Military Medical University, Chongqing 400038, China*

*^3^Key Laboratory of Tumor Immunopathology of Ministry of Education of China, Third Military Medical University, Chongqing 400038, China*

** Corresponding to: Yue Zhou and Bo Huang, Department of Orthopedics, Xinqiao Hospital, Third Military Medical University, Chongqing 400037, China*

Fax: +86 02368774328; Tel: +86 02368774328

E-mail address: happyzhou@vip.163.com, bighuang2008@hotmail.com

**Supplementary Information**

Supplemental Materials and Methods

**Proliferation (doubling time)**

To evaluate the doubling time, the morphology of CEP chondrocytes was checked by an inverted phase contrast microscope and their proliferation (growth curve) was measured by the DNA Hoechst 33528 dye stain assay (Sigma, USA). The fluorescence intensity with excitation at 365 nm and emission at 458 nm was read using a microplate reader (fluorescence mode, SpectraMax M5 Microplate Reader, Molecular Devices). Cell number was obtained from the value of intensity using a calibration curve. The cell doubling time was calculated based on the cell number collected from every group or time point.

**miRNA transfection**

Pre-miR-20a miRNA precursor, Anti-miR-20a miRNA inhibitor, Negative control were purchased from (Ambion, Applied Biosystems, USA). miR-20a was overexpressed by transfecting HEK293 or CEP chondrocytes with pre-miR-20a precursor every three days and the efficiency of transfection was confirmed by qPCR. In addition, the anti-miR-20a inhibitor was used to downregulate the expression of miR-20a in HEK293 or CEP chondrocytes. The pre-miR precursors, miRNA inhibitors and negative control molecules were transfected into cells using lipofectamine 2000 transfection reagent (life technologies, USA) according to the manufacturer’s instructions. Briefly, HEK293 or CEP chondrocytes were seeded at a density of 5 × 10^4^ cells/well. After the cells reached approximately 50% confluence, the pre-miR precursors, miRNA inhibitors and negative control were added into the walls. The final incubation concentrations of pre-miR precursors, miRNA inhibitors and negative control were all 20 nM. After a 6-h incubation, the culture medium in the wells was replaced by complete culture medium. Then, the cells were cultured for another 24 before being used for analyses.

**Real time polymerase chain reaction**

CEP chondrocytes cultured on PA gels with different stiffnesses and TCP were lysed at days 0, 7, 14 and 21 and total RNA was extracted from cells using RNeasy kit (QiagenGmbH, Germany) according to the manufacturer’s instructions. Isolated RNA was treated with RNase-free DNase (GiagenGmbH) before being reverse transcribed into cDNA. The purified RNA was quantified in a spectrophotometer (Beckman, Fullerton, CA) at 260 nm and 280 nm. The RNA was transcribed into cDNA with a ThermoScriptTM RT-PCR system (Invitrogen, USA) and amplified via PCR. Amplification primers were listed in Supplemental Table 1. The mRNA levels were normalized against the housekeeping gene glyceraldehydes-3-phosphate dehydrogenase (GAPDH).

**Western blotting**

Protein samples were separated on 10% (w/v) SDS gels and transferred onto PVDF membranes (Millipore, USA). The membranes were blocked with 5% milk proteins in TBST for 1 h at 37°C and then incubated with primary antibodies (ANKH, 1:1000 dilution (Santa Cruz, USA); Collagen I, 1:500 dilution (Sigma-Aldrich); Osteocalcin, 1:500 dilution (Sigma-Aldrich); GAPDH, 1:1000 dilution (Santa Cruz)) overnight at 4°C. The membranes were then incubated with HRP-conjugated anti-mouse secondary antibodies for 1 h at 37°C. Immunolabeling was detected using ECL reagent (Thermo Scientific, USA).

**Supplemental Tables**

**Table S1. Primer sequences for PCR**

| primers | Forward | Reverse | Tm(°) |
| --- | --- | --- | --- |
| ALPL | 5'-CTTGCTGGTGGAAGGAGGC-3' | 5'-AATGTGAAGACGTGGGAATGGT-3' | 59.9 |
| OCN | 5'-AGGGCAGCGAGGTAGTGAAGA-3' | 5'-CTAGACCGGGCCGTAGAAGC-3' | 61.5 |
| RUNX2 | 5'-TCCAGACCAGCAGCACTCCAT-3' | 5'-GCTTCCATCAGCGTCAACACC-3' | 62.7 |
| COL-I | 5'-CTGACCTTCCTGCGCCTGATGTCC-3' | 5'-GTCTGGGGCACCAACGTCCAAGGG-3' | 61.8 |
| ANKH | 5'-CCAGCAACAAACTGGTGAGC-3' | 5'-ACGAAACAGAGCGTGAGTGA-3' | 59.9 |

**Table S2. Patients information**

| **diagnosis** | **disc level** | **Modified Piffirman grade** | **Cartilage Endplate Degeneration grade** | **Gender** | **Age(year)** | **Stiffness(kPa)** |
| --- | --- | --- | --- | --- | --- | --- |
| **ismic spondylolysis** | **L4-L5** | **Grade2** | **2** | **F** | **14** | **30.9** |
| **ismic spondylolysis** | **L4-L5** | **Grade1** | **2** | **F** | **18** | **75.3** |
| **ismic spondylolysis** | **L5-S1** | **Grade2** | **2** | **M** | **21** | **71.3** |
| **lumbar fracture** | **L1-L2** | **Grade2** | **2** | **F** | **26** | **80.3** |
| **ismic spondylolysis** | **L5-S1** | **Grade2** | **2** | **F** | **22** | **60.7** |
| **thoracolumbar fracture** | **T12-L1** | **Grade3** | **2** | **M** | **28** | **101.4** |
| **thoracolumbar fracture** | **T12-L1** | **Grade2** | **2** | **M** | **25** | **68.3** |
| **ismic spondylolysis** | **L5-S1** | **Grade2** | **2** | **M** | **25** | **83.3** |
| **ismic spondylolysis** | **L5-S1** | **Grade3** | **2** | **F** | **19** | **178.3** |
| **ismic spondylolysis** | **L5-S1** | **Grade2** | **2** | **M** | **27** | **40.3** |
| **lumbar fracture** | **L1-L2** | **Grade1** | **2** | **M** | **26** | **98.3** |
| **lumbar fracture** | **L1-L2** | **Grade1** | **2** | **F** | **28** | **73.2** |
| **ismic spondylolysis** | **L5-S1** | **Grade2** | **2** | **M** | **30** | **71.5** |
| **ismic spondylolysis** | **L5-S1** | **Grade3** | **2** | **M** | **36** | **199.3** |
| **ismic spondylolysis** | **L4-L5** | **Grade3** | **4** | **M** | **24** | **512.7** |
| **ismic spondylolysis** | **L4-L5** | **Grade3** | **4** | **F** | **35** | **520.3** |
| **ismic spondylolysis** | **L5-S1** | **Grade2** | **4** | **M** | **32** | **510.2** |
| **ismic spondylolysis** | **L4-L5** | **Grade3** | **4** | **F** | **28** | **300.3** |
| **thoracolumbar fracture** | **L1-L2** | **Grade3** | **4** | **F** | **39** | **890.5** |
| **degenerative spondylolisthesis** | **L4-L5** | **Grade3** | **4** | **F** | **40** | **528.6** |
| **degenerative spondylolisthesis** | **L5-S1** | **Grade3** | **4** | **M** | **39** | **732.4** |
| **degenerative spondylolisthesis** | **L3-L4** | **Grade4** | **4** | **F** | **38** | **359.3** |
| **degenerative spondylolisthesis** | **L4-L5** | **Grade4** | **4** | **M** | **46** | **439.4** |
| **degenerative spondylolisthesis** | **L4-L5** | **Grade4** | **4** | **F** | **42** | **540.3** |
| **degenerative spondylolisthesis** | **L4-L5** | **Grade3** | **4** | **M** | **35** | **490.5** |
| **degenerative spondylolisthesis** | **L4-L5** | **Grade3** | **4** | **M** | **48** | **480.5** |
| **ismic spondylolysis** | **L5-S1** | **Grade4** | **4** | **F** | **47** | **512.8** |
| **ismic spondylolysis** | **L5-S1** | **Grade4** | **4** | **M** | **55** | **642.7** |
| **degenerative spondylolisthesis** | **L4-L5** | **Grade5** | **6** | **M** | **43** | **978.56** |
| **degenerative spondylolisthesis** | **L5-S1** | **Grade5** | **6** | **M** | **65** | **1262.5** |
| **lumbar canal stenosis** | **L4-L5** | **Grade5** | **6** | **F** | **52** | **790.4** |
| **lumbar canal stenosis** | **L3-L4** | **Grade4** | **6** | **F** | **49** | **752.8** |
| **lumbar canal stenosis** | **L4-L5** | **Grade6** | **6** | **M** | **58** | **783.7** |
| **lumbar canal stenosis** | **L5-S1** | **Grade5** | **6** | **M** | **54** | **1632.8** |
| **lumbar canal stenosis** | **L3-L4** | **Grade5** | **6** | **F** | **48** | **852.8** |
| **lumbar canal stenosis** | **L4-L5** | **Grade5** | **6** | **M** | **61** | **983.2** |
| **degenerative spondylolisthesis** | **L4-L5** | **Grade5** | **6** | **M** | **62** | **974.7** |
| **degenerative spondylolisthesis** | **L4-L5** | **Grade4** | **6** | **F** | **57** | **784.8** |
| **degenerative spondylolisthesis** | **L5-S1** | **Grade5** | **6** | **M** | **68** | **939.2** |
| **degenerative spondylolisthesis** | **L5-S1** | **Grade5** | **6** | **M** | **63** | **1028.3** |
| **degenerative spondylolisthesis** | **L4-L5** | **Grade5** | **6** | **F** | **56** | **1302.6** |
| **degenerative spondylolisthesis** | **L4-L5** | **Grade5** | **6** | **M** | **63** | **973.8** |
| **lumbar canal stenosis** | **L3-L4** | **Grade5** | **6** | **M** | **62** | **862.6** |
| **lumbar canal stenosis** | **L3-L4** | **Grade4** | **6** | **F** | **43** | **963.7** |
| **lumbar canal stenosis** | **L3-L4** | **Grade5** | **6** | **F** | **39** | **920.3** |
| **degenerative spondylolisthesis** | **L3-L4** | **Grade5** | **6** | **M** | **35** | **883.6** |
| **degenerative spondylolisthesis** | **L4-L5** | **Grade5** | **6** | **M** | **50** | **973.7** |
| **degenerative spondylolisthesis** | **L4-L5** | **Grade5** | **6** | **M** | **71** | **914.3** |

Stiffness represents the means of elastic modulus of each CEP sample.

**Supplemental Figures**

**
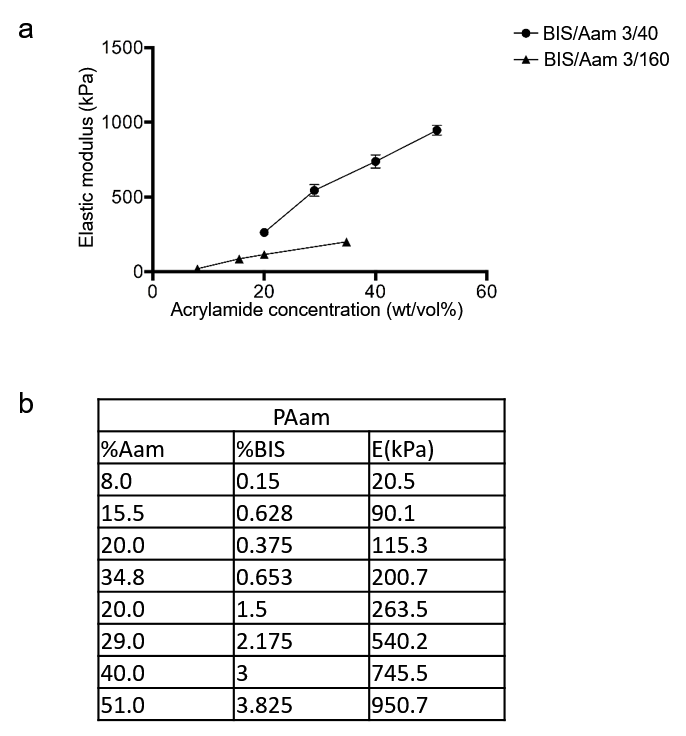
**

**Supplemental Figure1. Mechanical characterization of polyacrylamide gels.** a. Summary of polyacrylamide gels used. b. A table of the relative concentrations of acrylamide, bis-acrylamide and their desired and measured modulus of stiffness (average of 50 different measurements).


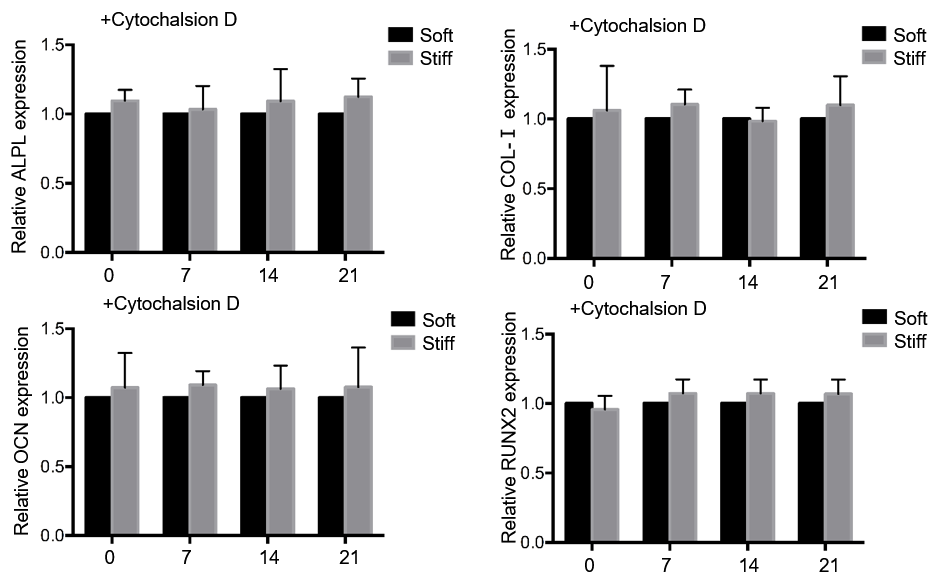


**Supplemental Figure 2. Reverse transcription (RT)-PCR analysis for the expression of RUNX2, ALPL, OCN and COL-I.** There was no significant difference in expression of calcification-related genes in CEP chondrocytes treated with CyD during all the time.


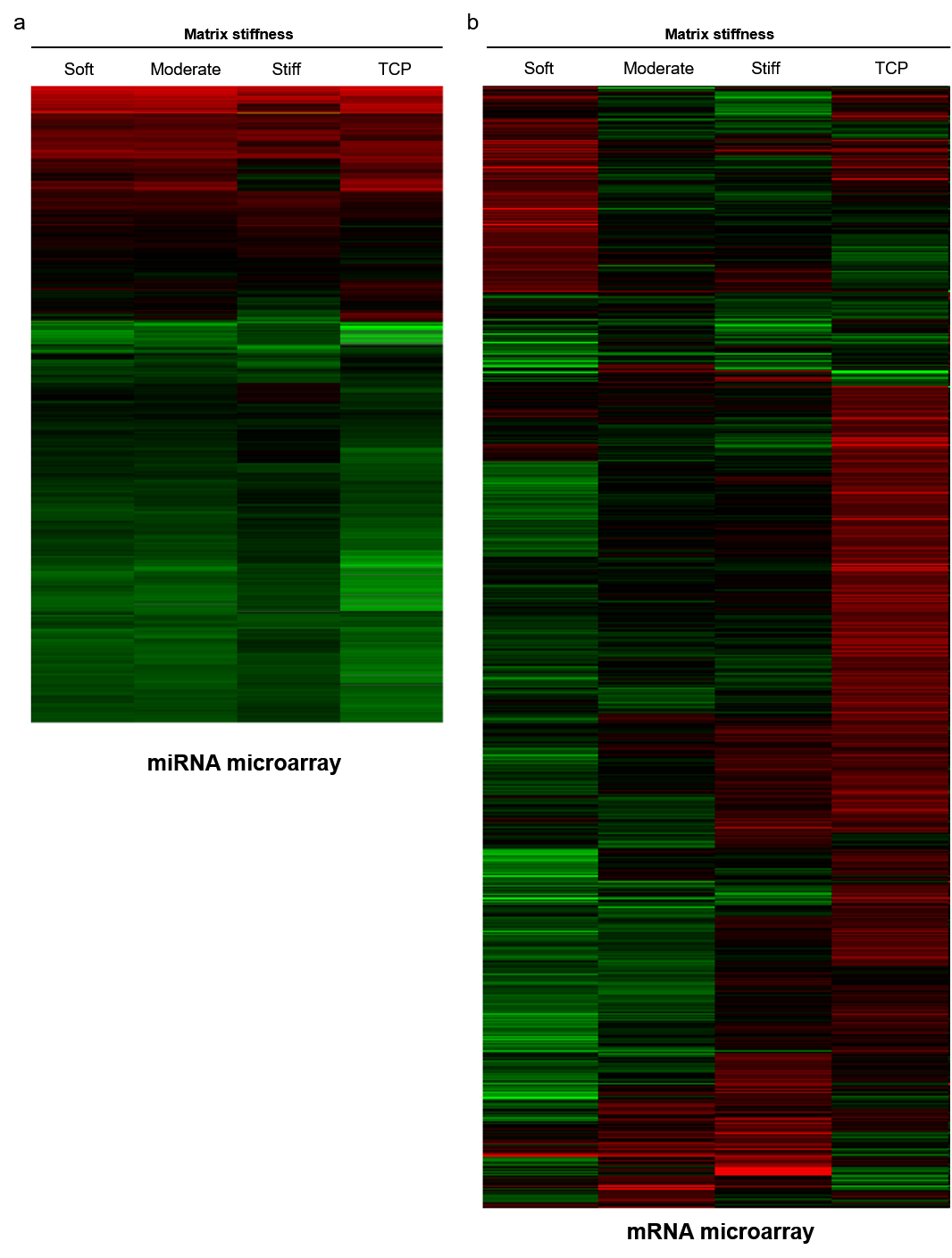


**Supplemental Figure 3. Hierarchical clustering results of the miRNA microarray and the mRNA.** CEP chondrocytes were cultured on PA gels of increasing stiffness (soft matrix, moderate stiffness, and stiff matrix respectively) and TCP with added Pi (3.0 mmol/L) for 14 days.

**
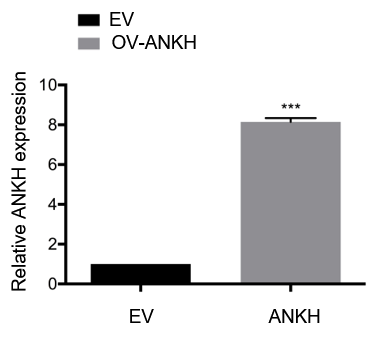
**

**Supplemental Figure 4. Real-time reverse transcription (RT)-PCR identified the overexpression of ANKH in CEP chondrocytes.** EV, empty vector; OV-ANKH, overexpression of ANKH by a retroviral vector.
